# Four-Year Trajectories of Internal Strengths and Socioemotional Support Among Middle-Aged and Older Adults with HIV

**DOI:** 10.1007/s10461-022-03798-z

**Published:** 2022-07-31

**Authors:** Lillian Ham, Bin Tang, Maulika Kohli, Dilip V. Jeste, Igor Grant, David J. Moore

**Affiliations:** 1 San Diego State University/University of California San Diego Joint Doctoral Program in Clinical Psychology, 220 Dickinson Street, San Diego, CA 92103 USA; 2 Department of Psychiatry, University of California San Diego, La Jolla, CA USA; 3 Department of Neurosciences, University of California San Diego, La Jolla, CA USA; 4 Sam and Rose Stein Institute for Research on Aging, University of California San Diego, La Jolla, CA USA; 5 HIV Neurobehavioral Research Program, San Diego, CA USA; 6 HIV Neurobehavioral Research Program, University of California San Diego, 220 Dickinson Street, Suite B (8231), San Diego, CA 92103 USA

**Keywords:** Positive psychology, Psychological resilience, Factor analysis, Chronic disease, Aging

## Abstract

Positive psychological attributes are associated with better health outcomes, yet few studies have identified their underlying constructs and none have examined their temporal trajectories in clinical vs. non-clinical samples. From data collected over 4 years from people with HIV (PWH) and HIV-uninfected (HIV−) participants, we identified two latent factors (internal strengths; socioemotional support) based on responses to seven positive psychological attributes. Internal strengths increased over 4 years for PWH, but not for HIV− comparisons. Socioemotional support did not change significantly in either group. Lower internal strengths and worse socioemotional support were related to greater depressive symptoms. We speculate that improvement in internal strengths in PWH could reflect their being in care, but this requires further study to include PWH not in care. Given the apparent malleability of internal strengths and their association with improved health outcomes, these attributes can serve as promising intervention targets for PWH.

## Introduction

As a result of the vast improvements in HIV treatment, more than half of people with HIV (PWH) in the United States are entering older adulthood (ages 50 years and above) [1]. PWH are at greater risk for neurocognitive impairment [2], medical comorbidities [3, 4] (e.g., cardiovascular disease), psychiatric comorbidities [5–8] (e.g., substance use, depression), and psychosocial stressors [9–12] (e.g., stigma) than the general population. Importantly, these biological and psychological factors negatively impact quality of life [13–16] and aging [17–19].

There has been a growing focus on positive psychology in the past two decades [20, 21]. In general and clinical populations, positive psychological attributes such as self-efficacy, optimism, resilience, and social support are associated with better health outcomes, successful aging, and higher quality of life [19, 22, 23]. Several studies on HIV have demonstrated that higher scores on these positive attributes predict slower HIV disease progression, better immune outcomes, and lower mortality [24]. Among adult PWH (20.5% < 40-years-old; 57.4% < 50-years-old), older age and shorter time diagnosed with HIV were associated with higher resilience and lower mental and physical health problems [25]. Moreover, positive psychological attributes may contribute to cognitive reserve, which has been defined as the adaptability of the brain that explains individual vulnerability to aging, pathology, or insult [26].

Whereas most HIV studies have used traditional measures of education, intelligence quotient (IQ), and socioeconomic status as proxies for cognitive reserve [27], more recent studies have used positive psychological attributes as well [17, 21–24]. Grit and ambition have been linked to better neurocognition and everyday functioning among middle-aged and older PWH, and may be protective against cognitive decline in this population [28]. Older PWH with higher self-rated successful aging also endorsed higher scores on seven positive psychological attributes (resilience, optimism, perceived stress, spirituality, social support, personal mastery, and attitudes toward own aging), and physical and emotional functioning [17]. Further, older PWH who were successful cognitive agers (defined as no neurocognitive impairment, no major depressive disorder, and no functional impairment) scored higher on ten positive psychological attributes (resilience, optimism, perceived stress, spirituality, social support, emotional support, personal mastery, post-traumatic growth, life satisfaction, and attitudes toward own aging) than PWH who were not, and scored comparably to HIV-negative (HIV−) participants [19].

While there is evidence for the benefits of positive psychological attributes among PWH, there are two remaining methodological limitations in the extant literature. First, studies have made broad conclusions based on various measures that reflect non-cognitive positive characteristics pertaining to personality, affect, belief systems, attitudes, and interpersonal relationships. The lack of consistent, common measures across studies limits the ability to compare findings and determine which positive attributes might serve as intervention targets. In addition, few studies have evaluated whether these attributes, many of which are correlated to some degree, reflect common or separate latent factors. A study examining correlates of frailty in middle-aged and older PWH and HIV− participants conducted a principal components analysis of nine psychosocial measures [29], in which two latent components were identified: grit, optimism, personal mastery, successful aging, depressive symptoms, perceived stress, and negative interactions comprised “positive resources/outlook”, and emotional and social support reflected “support by others”. In a study of older, HIV− women, Vahia et al. conducted a factor analysis of variables related to successful aging, which produced five factors. Of note, optimism, resilience, and self-efficacy comprised a “psychological protective factor,” while attitude towards own aging and a physical composite score comprised a “physical functioning factor” [30]. Moreover, some literature suggests that negative affect and depressive symptoms be considered separately from positive attributes. Though depression may dampen psychological resilience, prior work has demonstrated that adverse psychosocial constructs are not necessarily the opposite of positive ones [9, 23, 24, 31, 32]. Further investigation of latent factor structures would elucidate meaningful dimensions reflected by several positive psychological attributes.

Second, there is limited research on the longitudinal trajectories of such positive attributes among PWH, though the possible implications of this work have significant clinical relevance. A remaining question for this research area is whether to measure positive attributes at a single time-point or over time [24]. Malleable constructs that fluctuate over time (“state-like”) could be more promising targets for intervention than more stable constructs (“trait-like”). Some research has suggested that positive affect [31], quality of life [33], and social support fluctuate [33] over 1 year in middle-aged PWH; however, these studies are limited by their relatively restricted time frame, lack of HIV− comparisons, and general measures of positive and/or negative constructs.

Based on these gaps in the literature, the aims of the current study were twofold: (1) determine which latent constructs are being reflected in eight positive psychological attributes that have been associated with better health outcomes among PWH, and (2) investigate whether these constructs change over 4 years and differ by HIV status.

## Methods

### Participants

Participants were recruited from the *Multi-Dimensional Successful Aging Among HIV− Infected Adults*, which has been described in prior publications [13, 19, 29]. This longitudinal study was approved by the UC San Diego Institutional Review Board and conducted at the University of California, San Diego (UCSD) HIV Neurobehavioral Research Program (HNRP) and the UCSD Stein Institute for Research on Aging. Written informed consent was obtained from all participants after the study was explained to them by a trained staff member. Exclusion criteria included: acute drug or alcohol intoxication on day of testing (e.g., positive urine toxicology screen for illicit substances other than cannabis), significant neurological/neurodegenerative conditions unrelated to HIV (e.g., Alzheimer’s disease), and serious psychotic disorders (e.g., schizophrenia). All participants were assessed for comorbid medical conditions (e.g., self-reported presence of hepatitis C, hyperlipidemia, diabetes) and psychiatric conditions (e.g., major depressive disorder (MDD), substance use disorder). Inclusion criteria included: being ages 36–65-years-old, English fluency, and ability to provide informed consent.

Community-dwelling adults in San Diego and surrounding areas were considered for inclusion. Further exclusion criteria for the current study included: (1) only cross-sectional data and (2) missing ≥ 4 positive psychological attributes. Data were collected from 2013 to 2019 at baseline (Time 0) and at each subsequent annual visit over 4 years (Time 1–4). In total, 196 participants with complete baseline data (106 PWH, 90 HIV-negative participants) were retained for analyses.

### Measures

The primary variables of interest, eight positive psychological attributes, are described in Table 1. In addition, self-reported depressive symptoms were assessed via the 20-item Center for Epidemiologic Studies Depression Scale (CES-D) [34], which measures the frequency of depressive symptoms (e.g., “I felt that I could not shake off the blues even with help from my family”) in the past week using a 0–3 response format: 0—rarely or none of the time/less than 1 day to 3—all of the time/5–7 days. Higher scores reflect greater depression (total sum range 0–60). Diagnoses of current and lifetime MDD and substance use disorder were assessed via the Composite International Diagnostic Interview (CIDI) [35, 36], which follows DSM-IV diagnostic criteria.

**Table 1:** Positive psychological attributes

Measure (number of items)	Construct, *example item* (response format)	Directionality (score: range)
Grit Scale [37] (12-item short-form)	Grit (perseverance of long-term goals despite setbacks) e.g., “Setbacks don’t discourage me” (1-not like me at all to 5-very much like me)	Higher score = grittier (overall average: 1–5)
Philadelphia Geriatric Center Morale Scale [38] (5-item attitude towards own aging subscale)	Attitude towards aging (attitude towards and evaluation of the aging process one experiences) e.g., “I am as happy as when I was younger” (0-disagree and 1-agree)	Higher score = more positive attitude towards aging (sum: 0–5)
Life Orientation Test-Revised [39] (6-item overall resilience)	Optimism (individual differences in generalized optimism versus pessimism) e.g., “I’m always hopeful about my future” (1-strongly agree to 5-strongly agree)	Higher score = more optimism (sum: 6–30)
Satisfaction with Life Scale [40] (5-item)	Life satisfaction (global cognitive judgments of satisfaction with one’s life and participant well-being) e.g., “I am satisfied with my life” (1-not at all to 4-strongly agree)	Higher score = more satisfaction (sum: 5–35)
Pearlin Personal Mastery Scale [41] (7-item)	Personal mastery (self-concept and the extent to which one perceives themselves in control of forces that significantly impact their lives) e.g., “What happens to me in the future mostly depends on me” (1-strongly disagree to 4-strongly agree)	Higher score = more personal mastery (sum: 7–28)
Emotional Support Scale [42] (2-item emotional support subscale)	Emotional support (frequency and availability of emotional support) e.g., “How often are your spouse, children, close friends, and/or relatives willing to listen when you need to talk about your worries or problems?” (1-never to 4-frequently)	Higher score = more emotional support (average: 0–3)^a^
Duke Social Support Index [43] (4-item social interaction subscale)	Social support (number of close relationships and frequency of socialization) e.g., “About how often did you go to meetings or clubs, religious meetings, or other groups that you belong to in the past week?” (1-none to 3-seven or more times)	Higher score = more social support (sum: 4–12)
Brief Multidimensional Measure of Religiosity/Spirituality [44] (6-item daily spiritual experiences subscale, 5-item private religious practices subscale, 2-item overall self-ranking subscale)	Religiosity/spirituality (self-ranking) e.g., “To what extent do you consider yourself a religious person?” (1- very to 4-not religious at all)	Higher score = less religious/spiritual (sum: 13–81)

^a^Emotional Support Scale was administered with a response scale from 1 to 4, but the average score was calculated using a range from 0 to 3

### Statistical Analyses

Chi-square (${\chi }^{2}$) tests of independence, Fisher’s exact tests, Wilcoxon rank sum tests, and independent samples t-tests were conducted to compare the sample by HIV status and number of visits (Table 2; see section “Cohort Retention Over Time”). To address Aim 1, an exploratory factor analysis (EFA) with an orthogonal rotation (varimax) was conducted to identify latent factors among eight positive psychological attributes at baseline. The Kaiser–Meyer–Olkin (KMO) measure of sampling adequacy and Bartlett’s test of sphericity were used to check the appropriateness of conducting an EFA. Kaiser’s rule (eigenvalues > 1) and the scree plot were used to identify the number of factors. A factor loading cut-off of ≥ |0.4| was used to determine factor membership.

**Table 2:** Sociodemographic differences by HIV status at baseline

	Total (N = 196)	PWH (n = 106)	HIV− (n = 90)	Test statistic	Effect size
Demographics					
Age (years)	51.26 (8.03)	51.41 (8.35)	51.08 (7.67)	t = − 0.28	d = 0.04
Gender, N (% men)	151 (77.0)	90 (84.9)	61 (67.8)	${{\varvec{\chi}}}^{2}$ = 8.11**	V = 0.20
Race/ethnicity, N (%)				${{\varvec{\chi}}}^{2}$ = 9.65*	V = 0.21
White	117 (59.7)	57 (53.8)	60 (66.7)	–	–
Hispanic	36 (18.4)	19 (17.9)	17 (18.9)	–	–
Black	32 (16.3)	20 (18.9)	12 (13.3)	–	–
Asian	1 (0.5)	1 (1.0)	0 (0.0)	–	–
Other	10 (5.1)	9 (8.5)	1 (1.1)	–	–
Education (years)	14.60 (2.37)	14.16 (2.42)	15.11 (2.22)	t = 2.85**	d = 0.41
Comorbidities					
Hepatitis C, N (%)	24 (12.2)	20 (18.9)	4 (4.4)	${{\varvec{\chi}}}^{2}$ = 10.34**	V = 0.22
Hypertension, N (%)	65 (33.2)	49 (46.2)	16 (17.8)	${{\varvec{\chi}}}^{2}$ = 18.47***	V = 0.30
Hyperlipidemia, N (%)	63 (32.1)	43 (40.6)	20 (22.2)	${{\varvec{\chi}}}^{2}$ = 7.66**	V = 0.20
Diabetes, N (%)	18 (9.2)	11 (10.4)	7 (7.8)	${\chi }^{2}$ = 0.40	V = 0.05
Psychiatric characteristics					
Depressive sx (CES-D)	17.16 (7.1)	19.03 (8.11)	14.97 (4.83)	t = − 4.16***	d = 0.61
Current MDD, N (%)	13 (6.7)	13 (12.4)	0 (0.0)	${{\varvec{\chi}}}^{2}$ = 16.89***	V = 0.25
LT MDD, N (%)	118 (62.1)	54 (54.0)	18 (20.0)	${{\varvec{\chi}}}^{2}$ = 24.09***	V = 0.35
Current substance use dx, N (%)	5 (2.6)	4 (3.8)	1 (1.1)	${\chi }^{2}$ = 1.53	V = 0.09
LT substance use dx, N (%)	98 (51.6)	65 (65.0)	33 (36.7)	${{\varvec{\chi}}}^{2}$ = 15.43***	V = 0.28
HIV characteristics					
Current CD4 (c/μL)	–	637 [422–858]	–	–	–
Nadir CD4 (c/μL)	–	162 [29–319.8]	–	–	–
HIV duration (years)	–	17.31 (8.76)	–	–	–
On ART, N (%)	–	102 (97.1)	–	–	–
AIDS status, N (%)	–	67 (63.2)	–	–	–
Plasma viral load ≤ 50 (c/mL)	–	87 (91.6)	–	–	–
Positive psych. attributes					
Grit	3.73 (0.55)	3.59 (0.60)	3.90 (0.43)	t = 4.03***	d = 0.59
PGMS	3.56 (1.67)	3.00 (1.77)	4.26 (1.23)	t = 5.57***	d = 0.83
LOT-R	22.79 (4.68)	21.66 (4.85)	24.13 (4.10)	t = 3.81***	d = 0.55
SWLS	21.37 (7.49)	19.43 (7.39)	23.69 (6.97)	t = 4.10***	d = 0.59
PMS	22.29 (4.24)	21.29 (4.38)	23.50 (3.73)	t = 3.73***	d = 0.54
DSS	8.46 (1.80)	8.42 (1.91)	8.50 (1.65)	t = 0.29	d = 0.04
ESS	2.48 (0.71)	2.38 (0.78)	2.59 (0.61)	t = 2.04*	d = 0.30
BMMRS	51.31 (19.70)	49.88 (19.00)	53.06 (20.50)	t = 1.12	d = 0.16

PWH = people with HIV; sx = symptoms; CES*-*D = Center for Epidemiological Studies Depression Scale; LT = lifetime; MDD = major depressive disorder; dx = diagnosis; ART = antiretroviral therapy; psych*.* = psychological; PGMS = Philadelphia Geriatric Center Morale Scale; LOT*-*R = Life Orientation Test-Revised; SWLS = Satisfaction with Life Scale; PMS = Pearlin Personal Mastery Scale; DSS = Duke Social Support Index; ESS = Emotional Support Scale; BMMRS = Brief Multidimensional Measure of Religiosity/Spirituality (higher = less religious); d = Cohen’s d; V = Cramer’s V; *p*-values < 0.05 are bolded and denote statistical significance

*p < 0.05, **p < 0.01, ***p < 0.001. M (SD) or median [IQR] shown for continuous variables and count (%) for categorical variables

To address Aim 2, each positive psychological attribute was converted to a z-score using HIV− participants’ performance at baseline as a reference:

$$\frac{{{\text{Raw score at single timepoint}} - {\text{Average score for HIV- at baseline}}}}{{\text{SD for HIV- at baseline}}}$$

Based on the results of the EFA, respective groups of z-scores were averaged to create an overall average z-score that reflected a latent factor. Thus, each participant had unique latent factor z-scores at each timepoint. Linear mixed-effects models with subject-specific random intercepts and compound symmetry covariance structure were conducted to determine the effect of HIV status, time (years since baseline), and HIV status × time on each latent factor (overall average z-score). A non-significant interaction was removed from models. The following covariates were included in adjusted models if they were significantly related to the outcome in univariate analyses: age, White race, gender, education, hepatitis C, diabetes, hypertension, hyperlipidemia, and depressive symptoms (CES-D). Statistical analyses were performed in JMP® Pro 15.0.0 (SAS Institute Inc., 1989–2007) using two-tailed tests and a significance level of α = 0.05.

## Results

### Sample Characteristics

Numbers of participants (PWH, HIV−) at the timepoints from Time 0 to Time 4 were 196 (106, 90), 180 (93, 87), 164 (88, 76), 139 (76, 63), and 117 (71, 46), respectively. Baseline characteristics for the total sample and by HIV status are reported in Table 2 with effect sizes. PWH had a higher proportion of men and lower levels of education, in addition to a greater prevalence of self-reported comorbidities (i.e., hepatitis C, hypertension, hyperlipidemia), than HIV− participants. PWH also reported greater depressive symptoms and had higher incidences of MDD and lifetime substance use disorder. At baseline, PWH had lower scores on grit, attitudes towards aging, optimism, life satisfaction, personal mastery, and emotional support, but did not differ from HIV− participants in social support and religiosity/spirituality.

### Cohort Retention Over Time

Rates of attrition and missing data at each timepoint following baseline were as follows, Time 1: N = 180 (8.2%), Time 2: N = 164 (16.3%), Time 3: N = 139 (29.1%), and Time 4: N = 117 (40.3%). The Brief Multidimensional Measure of Religiosity/Spirituality (BMMRS) was excluded from further analyses due to its low association with any latent factor, leaving seven positive psychological attributes in the final analyses (see section “Aim 1: Exploratory factor Analysis of Positive Psychological Attributes”). Across participants and timepoints, there were 715 (89.8%) instances in which 0/7 psychological attributes were missing, 66 (8.3%) instances in which 1/7 were missing, 11 (1.0%) instances in which 2/7 were missing, and 4 (0.5%) instances in which 3/7 were missing. Of 196 participants, 31 (15.8%) had one or more consecutive visits excluded on the basis of missing data (≥ 4 missing attributes).

Associations between baseline characteristics (see Table 2) and total number of “complete” visits (defined as ≤ 3 missing attributes) were examined. Of 196 participants, 142 (72.4%) had a greater number of visits (defined as ≥ 4 visits). Participants with greater visits did not differ by HIV status (${\chi }^{2}$< 0.01, *p* = 0.948), but were more likely to be White (64.8% v. 46.3%, ${\chi }^{2}$ = 5.49, *p* = 0.019) and less religious/spiritual (M = 54.63 vs. 42.55, t = -3.94, *p* < 0.001) than participants with fewer visits. PWH with greater visits had a higher prevalence of HCV (23.4% vs. 6.9%, ${\chi }^{2}$ = 4.37, *p* = 0.037) compared to PWH with fewer visits. On the other hand, HIV− participants with greater visits had a higher prevalence of diabetes (20.0% v. 3.1%, ${\chi }^{2}$ = 6.31, *p* = 0.012) compared to HIV− participants with fewer visits.

With regards to HIV disease characteristics, PWH with greater visits had a higher prevalence of AIDS (74.0% vs. 34.5%, ${\chi }^{2}$ = 13.89, *p* < 0.001) and lower nadir CD4 counts (Median = 120 vs. 280, Z = 2.32, *p* = 0.021) than PWH with fewer visits. There were no other statistically significant differences comparing participants by number of visits and HIV status (*p*-values > 0.05).

### Aim 1: Exploratory Factor Analysis of Positive Psychological Attributes

The overall KMO for the current analysis was 0.86, which exceeds the generally recommended cut-off of 0.50 [45]. In other words, there was a high proportion of shared variance among positive psychological attributes. The Bartlett’s test of sphericity was statistically significant (${\chi }^{2}$(21) = 497.99, *p* < 0.001), supporting that the correlation matrix is factorable and that it is appropriate to proceed with the EFA. Using Kaiser’s rule and examining the scree plot suggested retaining two factors.

The EFA revealed two latent factors that accounted for 50.9% of the total variance (see Table 3). Using a factor loading cut-off ≥ |0.4|, attitude towards aging, optimism, personal mastery, life satisfaction, and grit loaded onto factor 1 (internal strengths), accounting for 32.5% of the total variance. Social and emotional support loaded onto factor 2 (socioemotional support), accounting for 18.5% of the variance. Religiosity and spirituality (higher BMMRS score = less religious/spiritual) had a rotated factor loading below |0.4| on internal strengths (− 0.15) and socioemotional support (− 0.17). A second EFA was conducted without BMMRS, which yielded two factors of the same membership. Internal strengths explained 38.0% of the variance and socioemotional support explained 19.6% of the variance, together accounting for 57.6% of the total variance. Due to its low factor loading, BMMRS was subsequently excluded from further analyses.

**Table 3:** Rotated factor loadings of exploratory factor analysis with orthogonal varimax rotation

Positive psych. attribute	Including BMMRS (total variance explained: 50.9%)		Excluding BMMRS (total variance explained: 57.6%)	
	Factor 1 (32.5% of variance explained)	Factor 2 (18.5% of variance explained)	Factor 1 (38.0% of variance explained)	Factor 2 (19.6% of variance explained)
Philadelphia Geriatric Center Morale Scale	**0.70**	0.32	**0.71**	0.29
Duke Social Support Index	0.08	**0.99**	0.12	**0.99**
Emotional Support Scale	0.34	**0.42**	0.35	**0.40**
Life Orientation Test-Revised	**0.75**	0.23	**0.75**	0.20
Personal Mastery Scale	**0.78**	0.23	**0.79**	0.20
Satisfaction with Life Scale	**0.68**	0.28	**0.69**	0.25
Grit	**0.58**	0.07	**0.58**	0.04
Brief Multidimensional Measure of Religiosity/Spirituality	− 0.15	− 0.17	–	–

The exploratory factor analysis excluding the Brief Multidimensional Measure of Religiosity/Spirituality was used in final analyses

psych*.* = psychological; BMMRS = Brief Multidimensional Measure of Religiosity/Spirituality (higher = lower religiosity/spirituality); bolded values are the factors that each attribute loaded most strongly onto for each solution

### Aim 2: The Effect of HIV, Time, and HIV × Time on Latent Factors

**Fig. 1:**
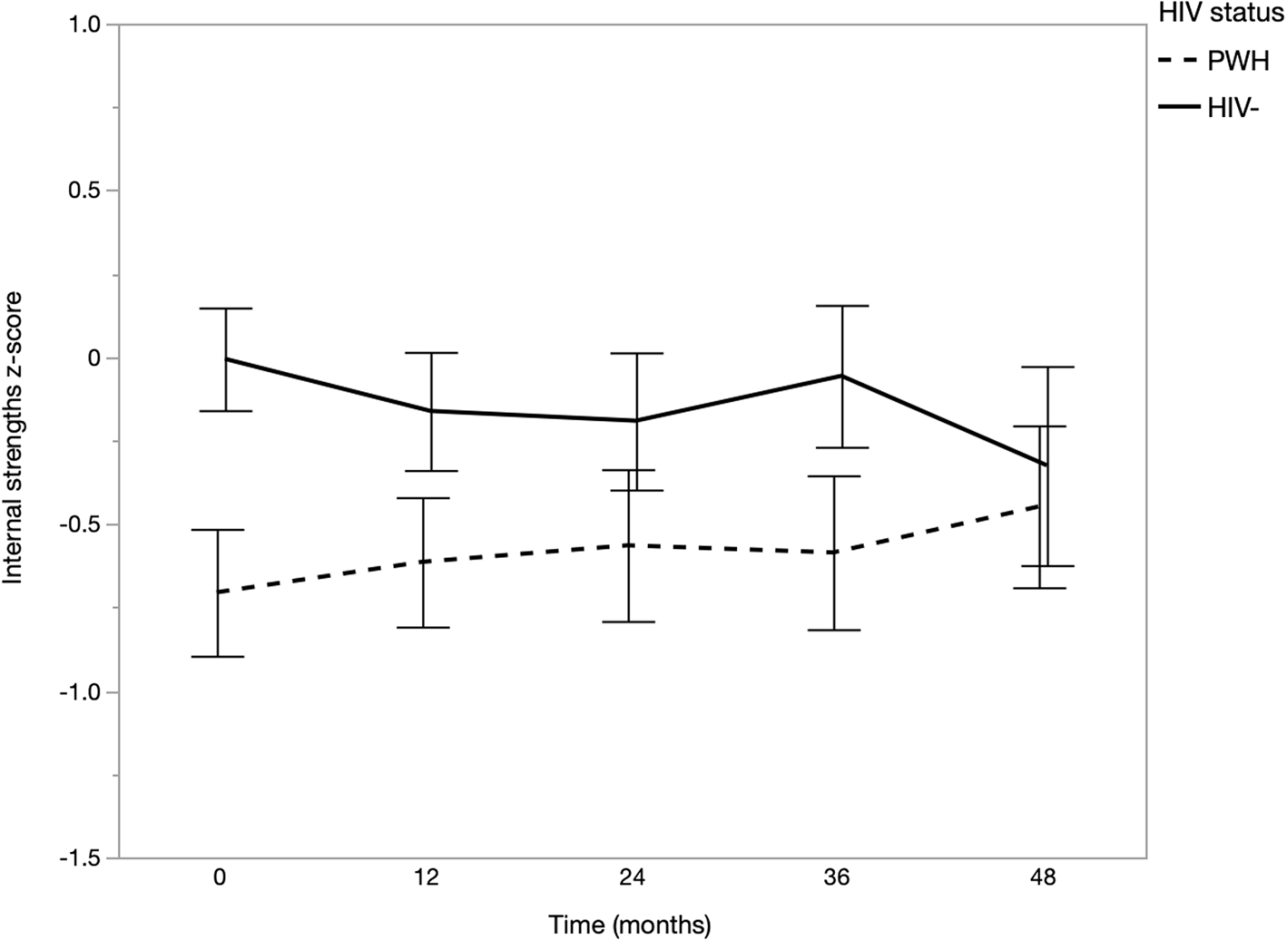
Means and 95% confidence intervals of internal strengths by time and HIV status. *p < 0.05, **p < 0.01, ***p < 0.001

**Fig. 2:**
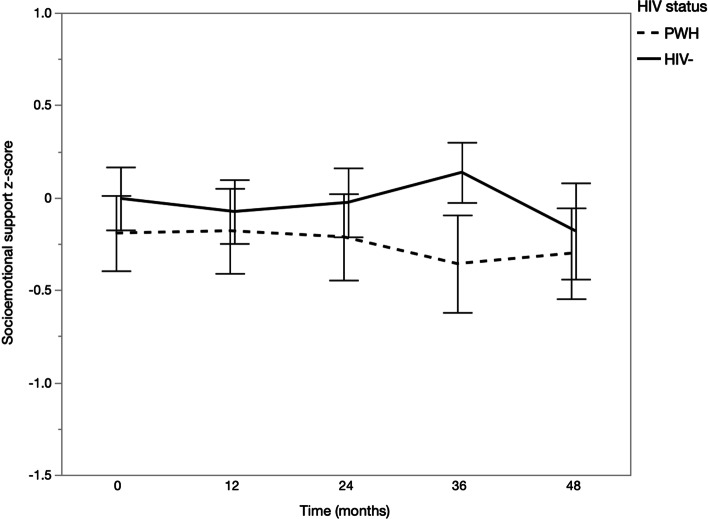
Means and 95% confidence intervals of socioemotional support by time and HIV status. **p < 0.01

**Fig. 3:**
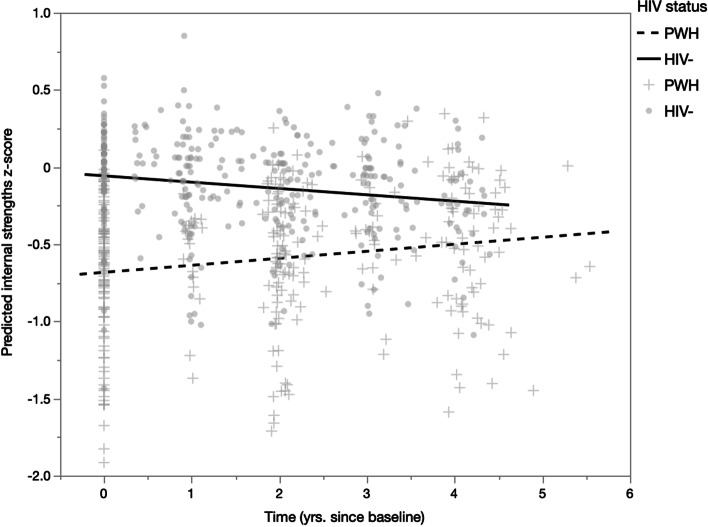
Predicted internal strengths z-score by time and HIV status, adjusting for covariates. Time significantly interacted with HIV status, B = 0.09, *p* < 0.001

HIV− participants had significantly greater internal strengths than PWH at all timepoints except at 48 months (Time 4), t = 2.39, *p* = 0.554 (see Fig. 1). In contrast, HIV− participants had greater socioemotional support than PWH at 36 months (Time 3) only, t = 2.99, *p* = 0.003 (see Fig. 2). Unadjusted linear mixed-effects models showed a significant HIV status × time (years since baseline) interaction effect on internal strengths, B = 0.06, *p* = 0.007 (see Table 4), which indicated that the change in internal strengths over time differed between PWH and HIV− individuals. This interaction remained significant after adjusting for White race, diabetes, hypertension, hyperlipidemia, and depressive symptoms, B = 0.09, *p* < 0.001 (see Table 4, Fig. 3). White participants (B = − 0.19, *p* = 0.041) and participants with hyperlipidemia (B = − 0.24, *p* = 0.011) had lower internal strengths than their respective counterparts. Further, greater depressive symptoms were associated with reduced internal strengths, B = − 0.05, *p* < 0.001. Simple effects analyses showed that for PWH, internal strengths significantly increased over time (B = 0.05, *p* = 0.005), whereas for HIV− participants, internal strengths had a decreasing trend over time (B = − 0.03, *p* = 0.071; see Fig. 3).

**Table 4:** Mixed effects models predicting internal strengths (overall average z-score)

	Unadjusted^a^			Adjusted		
	*B*	*SE*	*p*	*B*	*SE*	*p*
PWH Ref. HIV−	− 0.62	0.13	** < 0.001**	− 0.37	0.11	**0.002**
Time (yrs. since baseline)	− 0.04	0.02	**0.047**	− 0.03	0.02	0.071
HIV status × time	0.06	0.02	**0.007**	0.09	0.02	** < 0.001**
White race Ref. non-White	–	–	–	− 0.19	0.09	**0.041**
Diabetes Ref. absent	–	–	–	0.16	0.12	0.197
Hypertension Ref. absent	–	–	–	− 0.13	0.09	0.170
Hyperlipidemia Ref. absent	–	–	–	− 0.24	0.09	**0.011**
Depressive sx (CES-D)	–	–	–	− 0.05	4.43E−3	** < 0.001**

Covariance structure: compound symmetry; random effect: participant; *p*-values < 0.05 are bolded and denote statistical significance

PWH = people with HIV; yrs*.* = years; ref*.* = reference group; sx = symptoms; CES*-*D = Center For Epidemiological Studies Depression Scale

^a^Model without covariates

**Fig. 4:**
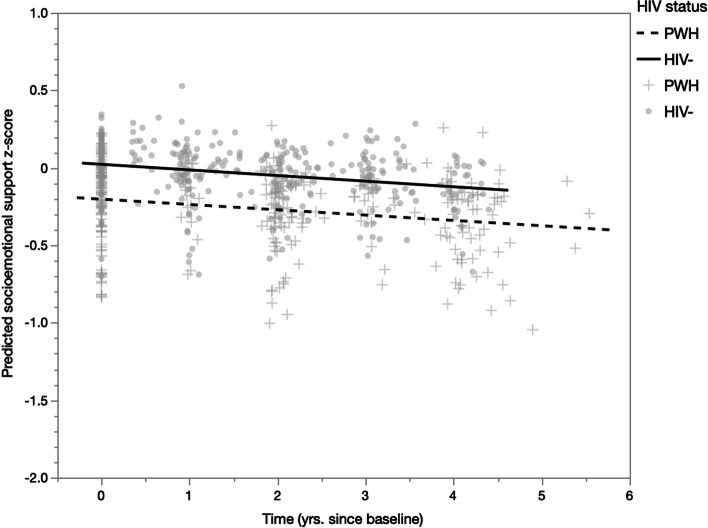
Predicted socioemotional support z-score by time and HIV status, adjusting for age, gender, hyperlipidemia, and depressive symptoms. Time was a non-significant predictor, B = − 0.03, *p* = 0.083

Unadjusted linear mixed-effects models did not show a significant HIV status × time interaction on socioemotional support, B = − 0.02, *p* = 0.492; therefore, the interaction was removed from the model. An unadjusted model with main effects of HIV status and time only showed a significant effect of time such that socioemotional support decreased for all participants, B = − 0.03, *p* = 0.020 (see Table 5). This effect did not remain significant after adjusting for age, gender, hyperlipidemia, and depressive symptoms, B = − 0.03, *p* = 0.083 (see Table 5, Fig. 4). Greater depressive symptoms were associated with reduced socioemotional support, B = − 0.03, *p* < 0.001.

**Table 5:** Mixed effects models predicting socioemotional support (overall average z-score)

	Unadjusted^a^			Adjusted		
	*B*	*SE*	*p*	*B*	*SE*	*p*
PWH Ref. HIV−	− 0.21	0.12	0.088	− 0.07	0.12	0.553
Time (yrs. since baseline)	− 0.03	0.01	**0.020**	− 0.03	0.02	0.083
Age	–	–	–	− 0.01	0.01	0.374
Men Ref. women	–	–	–	− 0.07	0.14	0.627
Hyperlipidemia Ref. absent	–	–	–	− 0.02	0.11	0.832
Depressive sx (CES-D)	–	–	–	− 0.03	0.01	** < 0.001**

Covariance structure: compound symmetry; random effect: participant; *p*-values < 0.05 are bolded and denote statistical significance

PWH = people with HIV; yrs*.* = years; ref*.* = reference group; sx = symptoms; CES*-*D = Center For Epidemiological Studies Depression Scale

^a^Model without covariates

## Discussion

In sum, the current study identified two latent factors that were reflected among seven positive psychological attributes: internal strengths (attitude towards aging, optimism, personal mastery, life satisfaction, and grit) and socioemotional support (social and emotional support). One rationale for this distinction may be that internal strengths are primarily driven by the self, whereas socioemotional support is driven by perceived interactions with others that are outside of one’s control. These findings support those reported by Rubtsova et al. [29], which similarly identified a construct reflective of positive resources/outlook and another construct representing support from others. A measure of religiosity/spirituality loaded poorly on internal strengths and socioemotional support, suggesting that this construct should be considered separately from the other psychological attributes.

At the group-level, internal strengths gradually increased over 4 years for PWH, but not for HIV− participants. A second factor representing socioemotional support did not change significantly in either group. Whereas PWH started with lower internal strengths than HIV-negative participants, PWH increased in internal strengths over 4 years such that internal strengths scores were comparable to that of HIV− participants at the final visit. These findings support that internal strengths are especially malleable among PWH and can even be improved. Depressive symptoms were negatively associated with both internal strengths and socioemotional support. PWH who were more likely to remain in the study had more advanced HIV (i.e., AIDS status, lower nadir CD4) and greater incidence of HCV. Broadly, participants who were White and less religious tended to have more complete data.

That PWH and not HIV− participants increase in internal strengths may suggest that internal strengths are protective and perhaps necessary to cope with a life-altering chronic illness [13]. On average, PWH in the current sample had HIV for 17 years (67% AIDS status). Receiving an HIV diagnosis can be a traumatic event that qualifies for HIV-related PTSD [46–48]. Factors such as internal strengths [19, 24, 49] and posttraumatic growth [50, 51] may facilitate positive coping behaviors to adjust to a life with HIV. Greater depressive symptoms and psychosocial distress [9] might attenuate this positive trajectory.

Continued involvement in research and engagement in the medical system might also reflect internal strengths such as grit and personal mastery, and may be particularly important for participants with more advanced HIV. In fact, the current study showed that participants with AIDS and lower nadir CD4 counts had more complete study visits than participants with less advanced HIV. It is also possible that engagement in care can improve well-being and self-efficacy. Future research comparing PWH who are in versus out of care would help determine if being in care itself benefits internal strengths.

These results support that internal strengths are promising treatment targets among PWH and that addressing one internal strengths attribute could likely generalize to other internal strengths. The developing literature on single (target one component of well-being) [52, 53] and multi-component positive psychology interventions (PPI; target at least two components of well-being) [54] have demonstrated efficacy for enhancing well-being and/or reducing psychological distress (i.e., depression, anxiety, stress) with small to moderate effects. Through this work, important theoretical frameworks on these mechanisms have been proposed. The current study’s findings align with the Synergistic Change Model [55], which posits that longer PPI gains can be achieved by targeting multiple domains of positive functioning and implementing a variety of individual activities (e.g., positive emotions, positive relationships, personal strengths) [54]. This outcome is achieved via decreases in risk of relapse and increases in probability of spill-over effects and synergy between activities.

Offering a PPI to both augment positive psychological attributes and alleviate symptoms of psychological distress may provide a promising alternate treatment for PWH. In fact, PPIs may be preferred among PWH in which psychiatric medication is contraindicated or undesired (e.g., PWH from backgrounds in which mental health stigma is pronounced). PPIs designed specifically for PWH have emerged more recently and are being conducted. These studies have shown promising results among newly diagnosed PWH [56], older PWH [57], and PWH who use methamphetamine [49], have depression [58], experience pain [59], and have low medication adherence [60]. In one acceptability study, the Transforming Lives Through Resilience Education program was tested among older PWH to boost resilience and mood, and improve outcomes related to aging and health behaviors [57]. In fact, PPIs that target internal strengths among older adults have shown to be particularly effective [53].

As the global prevalence of HIV-associated neurocognitive disorders (HAND) among PWH remains at nearly 45% [61, 62], future studies should consider how PPIs may contribute to better neurocognitive outcomes by maintaining cognitive reserve or reducing psychological distress (e.g., depression) and improving health behaviors (e.g., ART adherence). While neurocognition was not the focus in this study, the finding that internal strengths increased only for PWH may suggest that such attributes (e.g., grit) could play a greater role in maintaining neurocognition among PWH than in the general population [28]. Prior work has shown that older PWH who were successful cognitive agers similarly endorsed higher scores on internal strengths (optimism, personal mastery, attitudes toward own aging) and social support [19].

The current study investigated general, group-level changes in internal strengths and socioemotional support; however, unique trajectories of positive psychological attributes among subgroups of PWH have been observed in other studies [31, 33] and may identify who is more likely to improve in internal strengths and socioemotional support over time. Unsurprisingly, depressive symptoms were negatively associated with both factors and may dampen psychological resilience. Future longitudinal work is needed to identify profiles of trajectories and to investigate the relation between depression, internal strengths, and socioemotional support [13, 19].

This study is not without limitations. Post-hoc analyses showed that participants with fewer visits did not substantially differ on positive psychological attributes from participants with more visits; however, the total sample size was reduced from 196 at baseline to 117 at Time 4. Though linear mixed-effects models are typically robust to missing data, the effects of attrition and survival bias cannot be ruled out. Moreover, our sample of PWH consisted mostly of highly educated, White men. The generalizability of the current study is therefore limited as this sample is not entirely reflective of the population of PWH within the United States, which predominately consists of sexual minorities (gay and bisexual men), Black Americans, Hispanics/Latinos, and people who inject drugs [1]. Future studies with more diverse samples should investigate potential sociocultural differences among positive psychological attributes and the factors that contribute to these differences.

## Conclusions

We found that variables grouped under an umbrella of psychosocial constructs in prior studies comprise two latent factors: internal strengths and socioemotional support. Compared to HIV-uninfected individuals, internal strengths improved over time in people with HIV (PWH), suggesting that measures of internal strengths are malleable. Since our PWH were in care (97.1% on antiretroviral therapy), it is possible that engagement in healthcare improves internal strengths. Future studies including a comparison PWH group not engaged in regular care are needed. Given that better internal resources have been associated with improved health outcomes, these attributes may be an opportunity for positive psychological intervention activities.

## Data Availability

Contact corresponding author.
